# Effects of pH and Crosslinking Agent in the Evaluation of Hydrogels as Potential Nitrate-Controlled Release Systems

**DOI:** 10.3390/polym15051246

**Published:** 2023-02-28

**Authors:** María Dolores Ureña-Amate, María del Mar Socias-Viciana, María del Mar Urbano-Juan, María del Carmen García-Alcaraz

**Affiliations:** Department of Chemistry and Physics, Agroalimentary Campus of International Excellence (ceiA3), University of Almería, La Cañada de San Urbano, s/n, 04120 Almería, Spain

**Keywords:** hydrogels, controlled release, NMBA, EGDMA, column experiments, fertilizer

## Abstract

Water scarcity and the loss of fertilizer from agricultural soils through runoff, which also leads to contamination of other areas, are increasingly common problems in agriculture. To mitigate nitrate water pollution, the technology of controlled release formulations (CRFs) provides a promising alternative for improving the management of nutrient supply and decreasing environmental pollution while maintaining good quality and high crop yields. This study describes the influence of pH and crosslinking agent, ethylene glycol dimethacrylate (EGDMA) or N,N′-methylenebis (acrylamide) (NMBA), on the behavior of polymeric materials in swelling and nitrate release kinetics. The characterization of hydrogels and CRFs was performed by FTIR, SEM, and swelling properties. Kinetic results were adjusted to Fick, Schott, and a novel equation proposed by the authors. Fixed-bed experiments were carried out by using the NMBA systems, coconut fiber, and commercial KNO_3_. Results showed that on the one hand, no significant differences were observed in nitrate release kinetics for any system in the selected pH range, this fact allowing to apply these hydrogels to any type of soil. On the other hand, nitrate release from SLC-NMBA was found to be a slower and longer process versus commercial potassium nitrate. These features indicate that the NMBA polymeric system could potentially be applied as a controlled release fertilizer suitable for a wide variety of soil typologies.

## 1. Introduction

The current farming and agricultural production model are inadequate and insufficient to accomplish the Sustainable Development Goals (SDGs) agreed by the Food and Agriculture Organization of the United Nations in 2030 [1,2]. It is necessary to establish strategies of good agricultural practices to restore the nutrient deficit suffered by soils, increase crop yields, and minimize environmental impact, so it is essential to perform a responsible management of fertilizers and phytosanitary products, among others. Since plants do not fully use the quantities of conventional fertilizers that are applied to them, together with the increasingly high economic cost of raw materials and manufacturing costs, the consequent losses mainly due to the extensive use of nitrogen fertilizers are unaffordable [3,4].

Numerous strategies have been employed to mitigate the negative effects of this activity on the environment, including technology based on controlled release systems (CRS). The history of CRS dates to 1952 with Smith, Kline & French’s Spansule, a sustained release capsule technology that used granules with different dissolution rates to achieve release over 12 h [5]. However, in the mid to late 1980’s, the CRS field expanded to include drug-loaded, biodegradable polymer microparticles based on polyesters, these technologies having led to the creation of so-called nanomedicine [6,7]. The main effectiveness of these systems consists of an initial rapid release of the active ingredient, without complete release, which is subsequently slowed down to maintain a constant concentration of the active ingredient in the medium for the required period of time [8,9]. Depending on the nature of the stimuli, controlled release systems can respond to physical changes in temperature, light, electric or magnetic fields, and to chemical changes in pH, ionic strength, or the presence of chemical or biological compounds in the medium [10,11,12].

Therefore, there are significant opportunities for CRSs in agriculture to manage delivery of nutrients, such as nitrogen fertilizers, allowing a better targeting of the agrochemical, reducing costs, and improving environmental sustainability [13], this technology becoming a good alternative to traditional fertilization.

Hydrogels have become a very attractive type of materials to be used as controlled release systems in various branches of medicine, as well as in the field of agriculture where they have been used as soil amendments to improve soil hydraulic properties [13]. Hydrogels are three-dimensional systems of cross-linked polymer chains, of natural or synthetic origin, which in solution can absorb and contain large amounts of water molecules without dissolving or breaking their structure [14,15]. The absorption of such a large quantity of water, up to hundreds of times its size, is attributed to the presence of hydrophilic groups in the polymer chains, combined with the effects of osmotic pressure and capillarity, thereby achieving thermodynamic stability. The mentioned characteristics confer to these versatile materials properties such as elasticity, viscosity, low surface tension, or biodegradability [16,17]. The use of this type of material as a nitrate CRS could effectively regulate the amount of nitrogen added to the soil, while at the same time favoring water saving and contributing to improving the development of plants, thus improving the structure and aeration of soil. The properties of hydrogels are largely determined by the choice of crosslinking agent and other monomers that act as modifiers in the synthesis. Therefore, an increase in the concentration of the crosslinking agent results in a more compact macromolecular network, which improves its mechanical properties but decreases its water retention capacity [18].

Acrylamide and itaconic acid are two of the most widely used monomers in hydrogel synthesis [19,20,21,22]. Although both belong to the category of monomers with ionizable functional groups, the incorporation of itaconic acid to the cross-linked structure increases the hydrophilic character of the hydrogel, facilitating its use in numerous fields such as biomedicine, agriculture, pharmacy, or engineering [21,23,24,25,26]. Furthermore, the chemical structure of a hydrogel and the presence of ionizable functional groups in it significantly influences its swelling capacity, so a pH variation in the medium will have a significant influence on its behavior. On the contrary, if the hydrogel does not have any ionizable functional group, pH has no effect on swelling [27].

Therefore, the study of these types of polymeric materials in agriculture has aroused great interest in recent years, due to the enormous advantages they present such as low cost, acceptable specific resistance, good thermal properties, recyclability, etc. [28,29].

The aim of this study is the synthesis and characterization of polymeric materials based on itaconic acid and acrylamide, using ethylene glycol dimethacrylate (EGDMA) or N,N′-methylenebis(acrylamide) (NMBA) as crosslinking agents. The behavior of the polymeric systems against pH variation in the swelling process and in the release of nitrate ions, in both batch and dynamic regimes, has been evaluated.

## 2. Materials and Methods

### 2.1. Materials

The copolymers used in this work were synthesized from the following reagents supplied by Sigma-Aldrich: monomers (itaconic acid and acrylamide), crosslinkers (ethylene glycol dimethacrylate and N,N′-methylene-bis-acrylamide), redox initiators (potassium persulphate and sodium bisulfite). The fertilizer used in release studies was KNO_3_ (Panreac). The solvent used was distilled water and the media in swelling and release studies was Milli-Q water. A Britton–Robinson buffer solution was used to study the influence of pH on both the swelling process and the release of nitrate ions. A 0.04 M Britton–Robinson solution with respect to orthoboric, orthophosphoric, and acetic acids (Panreac) was prepared. This solution is very acidic (pH = 2), so sodium hydroxide (1 M, NaOH, 98%, Panreac) was used to increase its alkalinity. All reagents were of analytical grade and used without further purification.

### 2.2. Polymeric Systems

The polymeric systems (PS) used in this work were formulated using the copolymerization method described in Urbano Juan et al. [30]. Briefly, four hydrogels of different composition were synthesized by varying the type of crosslinker (EGDMA or NMBA), with or without fertilizer in the preparation (2.5 g KNO_3_). Aqueous solutions of the components were prepared by dissolving itaconic acid, acrylamide, the corresponding crosslinker, and the redox initiators in distilled water under constant stirring at room temperature. For those systems that contain the fertilizer, potassium nitrate was dissolved in distilled water before adding the rest of the components to this aqueous solution; thus, the active ingredient is uniformly included in the whole structure when the polymers network has been formed. The reaction mixtures were homogenized by ultrasound and degassed with nitrogen. Aliquots of 0.20 mL were placed in Eppendorf tubes as polymerization reactors and dried in oven at 60 °C for 24 h to obtain the xerogel. The hydrogels without nitrate (H-systems) are labelled as H-EGDMA and H-NMBA, and the ones containing nitrate (SLC-systems)—SLC-EGDMA and SLC-NMBA. All samples obtained were of conical geometry and glassy aspect, except for those synthesized with KNO_3_, which presented a whitish appearance.

#### Structural Characterization of the Polymeric Systems

The spectroscopic characterization of the xerogels with or without nutrient was performed through Fourier Transform Infrared Spectroscopy (FTIR). The samples were pounded and completely mixed with FTIR grade potassium bromide (Sigma-Aldrich, Madrid, Spain). The spectra of the pressed tablets were recorded with an Alpha I Bruker spectrometer at room temperature) with a resolution of 4 cm^−1^ and were averaged over 32 scans.

The morphological analysis was carried out by using a scanning electron microscope (SEM). In order to observe structural changes generated in the PS after the hydration process, aerogel samples were prepared using the critical point drying (CPD) technique [31,32]. In order to do this, the sample was subjected to a increasing consecutive dehydration process, by using six hydro-alcoholic mixtures containing 50 mL of distilled water:ethanol at different concentrations. The concentration gradient of these hydro-alcoholic mixtures was 10%, 30%, 50%, 70%, 85%, and 100%, all prepared with 96° ethanol (C_2_H_6_O, Panreac), except for the 100% mixture, which was prepared with absolute ethanol. The latter solution was washed twice consecutively to ensure complete removal of water. Then, the sample was placed in a Bal-Tec CPD 030 equipment where the absolute ethanol was displaced by diffusion by 10 washes with liquid CO_2_ at a temperature of 6–7 °C for 10 min and subsequently evaporated at 40 °C. SEM images of all samples (xerogels and aerogels) were visualized with a S-3500N Hitachi scanning electron microscope with a thermionic cannon of the wolfram forked filament type.

### 2.3. Effect of pH on Swelling Kinetics

To study the influence of pH on the swelling process of H-systems, the gravimetric method was followed and 0.04 M Britton–Robinson buffer solutions at pH 2, 4, 6, 8, and 10 were used as absorption medium. Flasks containing 100 mL of Britton–Robinson solution and xerogel samples were placed in a thermostatic bath at 25 °C with constant agitation (150 rpm). At different time intervals, swollen samples were removed, wiped with filter paper, weighed, and placed back into the flasks. This procedure was repeated until a constant mass was reached for each sample. All tests were carried out in duplicate.

The same assay described above was carried out with the SLC-systems, although in this case, the study was only conducted at pH 6, 8, and 10, as this covers the range of pH above and below that is usually found in agricultural soils.

The swelling percentages (S_w_) of the systems were calculated from the following equation:

S_w_ = (W_w_ − W_d_/W_d_) × 100 (1)

where W_w_ is the wet weight of the H/SLC-systems and W_d_ is the dry weight of the H/SLC- systems before swelling (xerogel).

Finally, taking into account that the initial swelling velocity (V_i_) is the maximum degree of swelling reached by the hydrogel during its initial stage of linear swelling over time (Equation (2)):

V_i_ = S_w_/t (2)

### 2.4. Effect of pH on Nitrate Ion Release Kinetics

Nitrate release from SLC-systems was determined by placing each xerogel in a flask containing 100 mL of Britton–Robinson buffer solution at the selected pH and placed into the thermostatic bath at the conditions indicated above (150 rpm, 25 °C). At different fixed time intervals, two aliquots of the release medium were taken for further analysis by ionic chromatography. This procedure was repeated until a constant concentration was reached for each sample.

The concentration of nitrate ions was determined by ionic chromatography using a DX-120 Vertex Technics S.L. equipment (Barcelona, Spain). Separation was performed on an AS9-HC 4 × 250 mm Dionex IonPac analytical column. The mobile phase was a 9 mM solution of Na_2_CO_3_ and an ASRS ULTRA II-4 mm Dionex was used as suppressed column.

### 2.5. Diffusion Mechanisms

To determine the nature of the absorbed water molecules diffusion process and nitrate ions that are released into the medium, the data from the kinetic studies carried out at pH 6, 8, and 10 were fitted both to first-order kinetics [33] in the first four hours of the tests and to second-order kinetics (Schott model) in the complete process.

The Ritger-Peppas equation (Equation (3)) for small molecules, based on Fick’s laws, allows to quantitatively determine the particle flux that occurs during the concentration gradient of a substance [33,34,35,36,37,38].

(3) $$\frac{M_{t}}{M_{\infty }}=k\cdot t^{n}$$

where M_t_ and M_∞_ are the amounts of the absorbed medium or the released fertilizer at a time t and at equilibrium, respectively, n is the diffusional exponent, which determines the type of mechanism, and k is the constant of the fertilizer–polymer system that depends on the structural and geometric characteristics of the hydrogel. Note that the fourth hour of the tests has been considered as the equilibrium time, as this equation only applies at the initial stages of the swelling and release processes, as well as in the case of SLCs when 60% of the fertilizer has been released.

The values of the diffusional exponent (n) delimit the different types of transport mechanism (Table 1) [34].

The diffusion coefficient (D) has been calculated using an equation proposed by the present authors, which is an adaptation of the equation proposed by Ritger–Peppas, thus allowing its application to any type of diffusion mechanism in hydrogels of conical geometry [30,39,40] (Equation (4)).

(4) $$\frac{M_{t}}{M_{\infty }}=4\cdot {\left[\frac{D}{\pi^{2}\cdot r\cdot \left(g+r\right)}\right]}^{n}\cdot t^{n}$$

where r and g are the radius and the generatrix of the xerogel of conical geometry, respectively.

Schott’s model, in contrast to the previous model, describes the diffusion process from a global perspective considering that in the initial state, diffusion occurs at a higher rate and subsequently slows down progressively until a constant final state is reached and maintained.

The equation describing this model, corresponding to second order kinetics, takes into account not only that the rate of diffusion of the species is directly proportional to the swelling percentage or release of nitrate ions that has not yet taken place at a given time, but also to the total internal area of the polymer system where no process occurred for the same time [41,42].

The equation proposed by Schott [41,42] (Equation (5)), describing the overall behavior of the diffusion process is given by the following equation [20,37,41,42,43,44]:

(5) $$\frac{t}{C_{t}}=\frac{1}{K_{\mathrm{ap}}}+\frac{t}{C_{e}}$$

where C_t_ and C_e_ are the quantities of medium absorbed or fertilizer released at time t and equilibrium, respectively, and K_ap_, the equilibrium apparent rate constant.

### 2.6. Study of Nitrate Ion Release in Dynamic Regime

**Figure 1:**
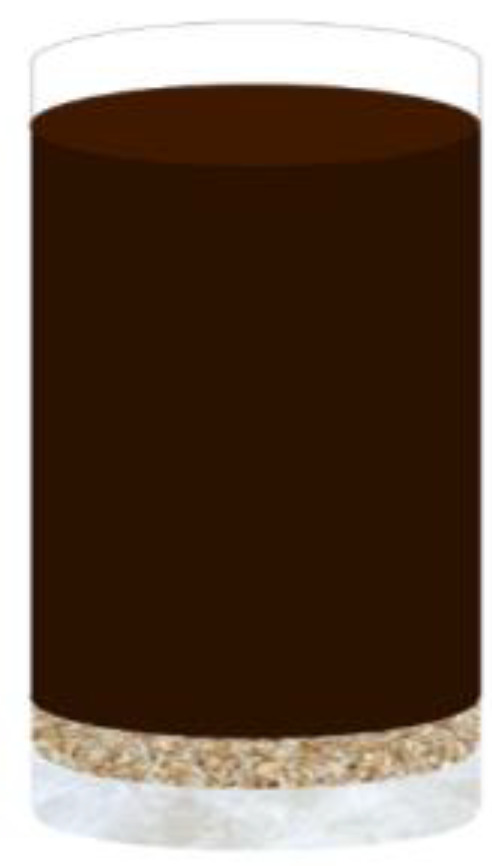
Schematic illustration of a coconut fiber column.

The study of the dynamic behavior was carried out in columns containing coconut fibre (Pelemix España, Murcia, Spain) as follows: PVC tubes (130 mm long and 55 mm in i.d.) were sealed in the base of each column with nylon mesh with an effective pore diameter of 60 mm, minimizing the dead-end volume. Then, a thin layer of 1 cm thick glass wool was placed at the bottom, on top of which 45 g of acid-washed sand (SiO_2_, Sigma-Aldrich, Madrid, Spain) was added to avoid any loss of filtering bed particles. Each column was filled with 45 g of coconut fiber. The addition was made in small portions, trying to homogenize its distribution within the column to achieve a bed height of 10 cm (Figure 1).

The pore volume (Vp) was estimated by calculating the difference in weight between the saturated columns after the excess water had been drained and the weight of the dry column. The Vp was added at a constant flow rate (6 mL/min) by using a Gilson peristaltic pump, model Minipuls3 at intervals of 24 h.

All studies were performed in duplicate. Previously, the columns were immersed in distilled water until saturation and left to leach for 15 min. To carry out the swelling and nitrate ion release experiments, a sample of the corresponding xerogel was placed at a depth of 3 cm and three types of tests were performed with different purposes:

T-0 test

(i) Analysis of the nitrate ion content in the coconut fiber

For this study, 4 Vp of distilled water were passed through each column at a constant flow rate.

(ii) Analysis of the nitrate ion retention capacity of the coconut fiber

One Vp of a potassium nitrate solution (100 mg NO_3_^−^/L) was added to the column, followed by the addition of 3 Vp of distilled water for studying the leaching of nitrate ions.

T-1 test

The aim of this test is to compare the swelling capacity of H-systems and SLC-systems under dynamic conditions. For this purpose, one xerogel was placed in each column and then 4 Vp of distilled water were added.

T-2 test

The purpose of these experiments was to compare the release of nitrate ions contained in the polymeric network of SLCs and commercial product, KNO_3_. For that, columns containing a xerogel and the commercial KNO_3_ (0.0399 g) were prepared, respectively, and placed at a 3 cm depth. Then, 4 Vp of distilled water was passed over them.

## 3. Results and Discussion

### 3.1. Structural Characterization of the Polymeric Systems

The FTIR spectra and the SEM images of all xerogels have been reported in Urbano Juan et al. (2019) [30]. In these spectra, the presence of absorption bands characteristic of these materials was observed such as those corresponding to the O-H and amide N-H stretching vibrations (3450 cm^−1^) or the band corresponding to the carboxylic groups and amides C=O stretching vibrations (1660 cm^−1^–1640 cm^−1^). On the other hand, the presence of the band at 1380 cm^−1^ confirmed the incorporation of the nitrate ion into the SLC-systems [20,45,46].

In relation with the SEM images obtained from xerogel samples, the presence of interconnected pores in their structures was observed, which confers them as big capillarity, converting them into superabsorbent materials [31,32]. The presence of small KNO_3_ crystals adhered to the surface of the SLC-systems could be also observed.

**Figure 2:**
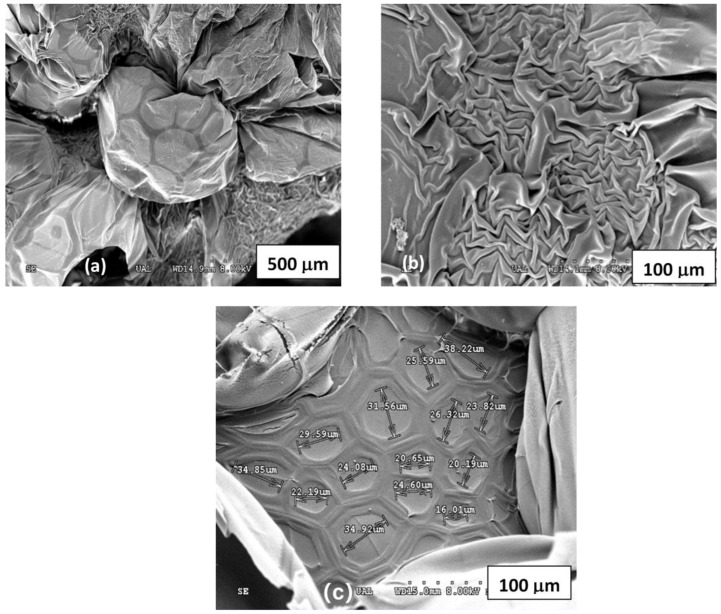
SEM images of H-NMBA system: (**a**) vesicles, (**b**) folds, and (**c**) marks of the interconnected cavities.

Figure 2 shows different micrographs obtained for the H-NMBA system in aerogel phase.

In these images, vesicles, surface folding, as well as traces and marks of the interconnected channels can be observed, which allow us to confirm that the internal structure of these hydrogels is of a mesh type, in which the cavities present hexagonal geometry, and whose sizes range between 16 µm and 38 µm. It should be noted that it was not possible to obtain the micrograph of H-EGDMA in the aerogel state, as it was completely dissolved in the hydro-alcoholic dehydration stage.

**Figure 3:**
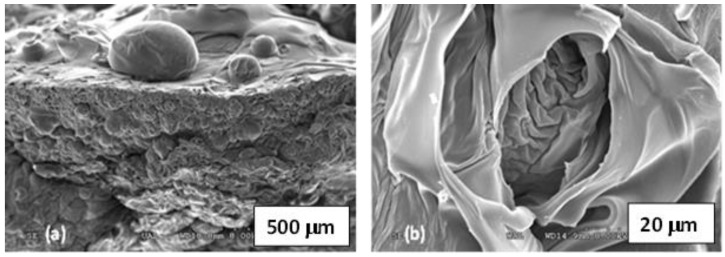
SEM images in aerogel SLC- systems: (**a**) SLC-NMBA, (**b**) SLC-EGDMA.

Figure 3 shows the images corresponding to the SLC-EGDMA and SLC-NMBA aerogel samples.

The NMBA-containing aerogel (Figure 3a) shows numerous small vesicles compared to those observed for the SLC-EGDMA system, where the presence of broken vesicles allows to see the large folds inside the polymeric structure (Figure 3b). As can be observed, any potassium nitrate crystals adhered to the surface are present due to the washing given to the samples during the hydro-alcoholic treatment.

### 3.2. Effect of pH on Swelling Kinetics

#### 3.2.1. H-Systems

**Figure 4:**
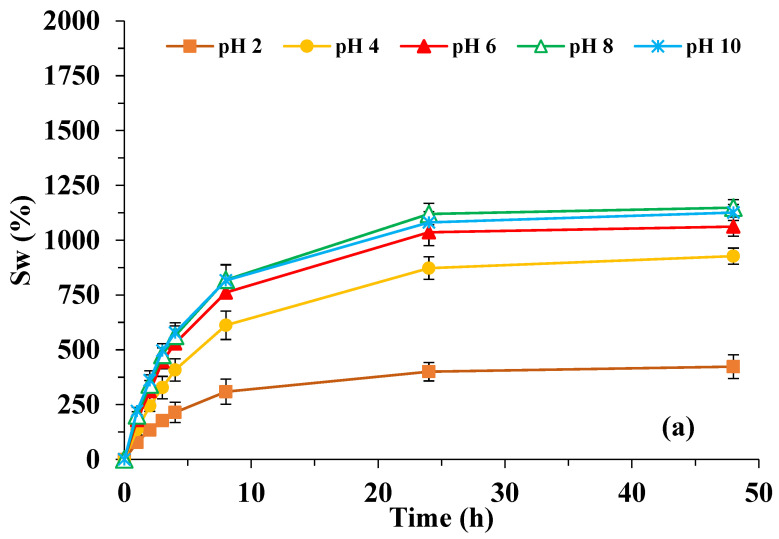

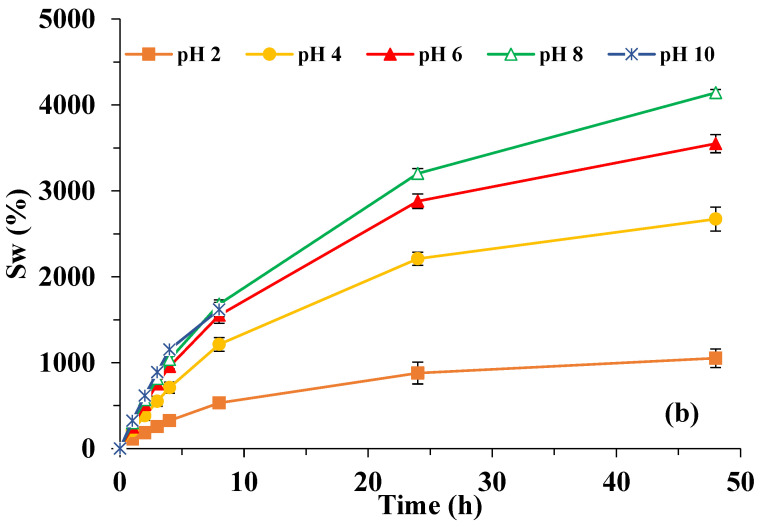
Swelling kinetic curves for (**a**) H-NMBA and (**b**) H-EGDMA systems at different pH.

The analysis of Figure 4 shows remarkable differences in the behavior of the synthesized polymeric materials. On the one hand, the H-EGDMA systems (Figure 4b) do not reach equilibrium at 48 h, and, in addition, they show much higher swelling capacities than the H-NMBA systems (Figure 4a) regardless of pH, the swelling equilibrium being reached for the latter systems after 24 h. These differences can be justified if we take into account that the systems containing EGDMA show a lower degree of crosslinking due to their less hydrophilic character and that the presence of branches in their molecule gives rise to a greater opening to the cavities of the three-dimensional network.

The joint action of these two facts allows the entry of a greater number of water molecules compared to systems containing NMBA. For this crosslinking agent, the presence of the NH groups facilitates the interaction with the hydrophilic groups of both itaconic acid and acrylamide, generating additional crosslinks through the formation of hydrogen bonds. The higher number of crosslinks makes the structure more rigid, closed, and therefore with a lower absorption capacity [30,47].

In relation to the influence of pH, Table 2 shows the swelling data determined at 48 h (S_w_), as well as the values of the initial linear velocities of swelling (V_i_). As can be seen, the S_w_ values increased as the pH of the medium increased, with the largest increase observed when the pH was changed from 2 to 4.

Taking into account that these PS contain itaconic acid (pK_a1_ = 3.85 and pK_a2_ = 5.44), when the pH values are above the pK_a_, electrostatic repulsions appear between the carboxylate groups, increasing the distance between the chains and causing an increase in their swelling capacity. It is evident that, as the pH raises, the ionization degree will increase and will give rise to greater repulsions between the polymeric chains, so that the hydrogel will swell more until it reaches 100% ionization. At this point, ionization will stabilize, as well as its swelling capacity [9,24,48].

The low increments in S_w_ from pH 6 for H-NMBA could be justified by two reasons: (i) the greater rigidity of the network, and (ii) the lower number of groups susceptible to ionization due to their participation in the crosslinking, therefore meaning this system had practically reached its maximum ionization percentage. On the contrary, the lower degree of cross-linking of the H-EGDMA network provides a high number of carboxylic groups susceptible to ionization at higher pH values. This explains the high increases in S_w_ when the pH of the medium rises from 6 to 8. However, it should be noted that the S_w_ value at pH 10 for H-EGDMA is not shown in Table 2 because it was completely dissolved 8 h after the beginning of the swelling phase (salting-in effect) [30,49,50].

As can be observed, V_i_ values obtained for the H-EGDMA system are practically double those corresponding to the H-NMBA that increase as the pH does. This confirms, on the one hand, the greater openness of the network of the H-EGDMA systems and, on the other hand, that as the pH increases, the repulsion effect between the carboxylate groups facilitates the entry of water molecules into the structure. Finally, the similarity of the V_i_ values from pH 6 onwards in the H-NMBA, corroborates that the carboxylic groups of these systems have reached their maximum ionization percentage.

#### 3.2.2. SLC Systems

**Figure 5:**
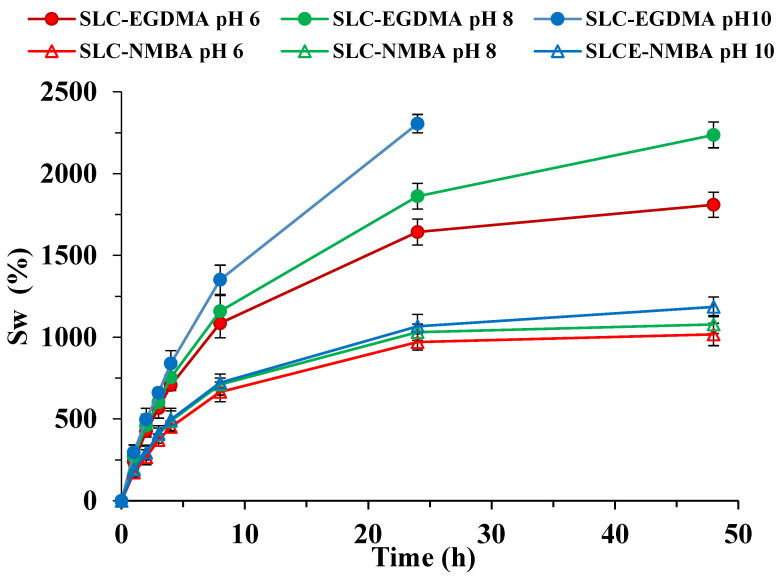
Swelling kinetic curves for SLC systems at pH 6, 8, and 10.

Figure 5 shows the swelling kinetics curves obtained for the SLC systems in the pH range 6 to 10, and the corresponding S_w_ and V_i_ parameters are given in Table 3.

The analysis of the swelling kinetics curves of these materials revealed a similar behavior to that indicated for the H-systems, with a first quick swelling phase up to approximately 8 h, followed by a second phase where the velocity decreases and reaches a plateau in the systems containing NMBA, in contrast to those with EGDMA.

Data in Table 3 indicate that, as occurred for H-systems, although less pronounced, S_w_ values are higher for the systems containing EGDMA. The comparison between S_w_ values for both H-systems (Table 2) and SLC-systems (Table 3) highlight the strong influence of the type of crosslinking agent. Thus, whereas the H-NMBA S_w_ values are very similar to their corresponding SLCs, the H-EGDMA S_w_ values are almost twice as high as those obtained for SLC-EGDMA systems. In this latter case, the nitrate and potassium ions inside the cavities of the structure not only compete with the water molecules for the most hydrophilic functional groups of the polymer chains, but also shield the electrostatic repulsions between the functional groups of the polymer chains, decreasing the distance between them and closing the structure [19,51,52].

In relation to the influence of pH, and in the same way as described above, formulations containing EGDMA showed the higher increases in S_w_ values with increasing pH. Again, the dissolution of these materials occurred after 24 h due to the salting-in effect at pH 10.

Finally, the V_i_ values confirm the behavior indicated above, with the SLC-EGDMA presenting the highest velocities.

### 3.3. Effect of pH on Nitrate Ion Release Kinetics

**Figure 6:**
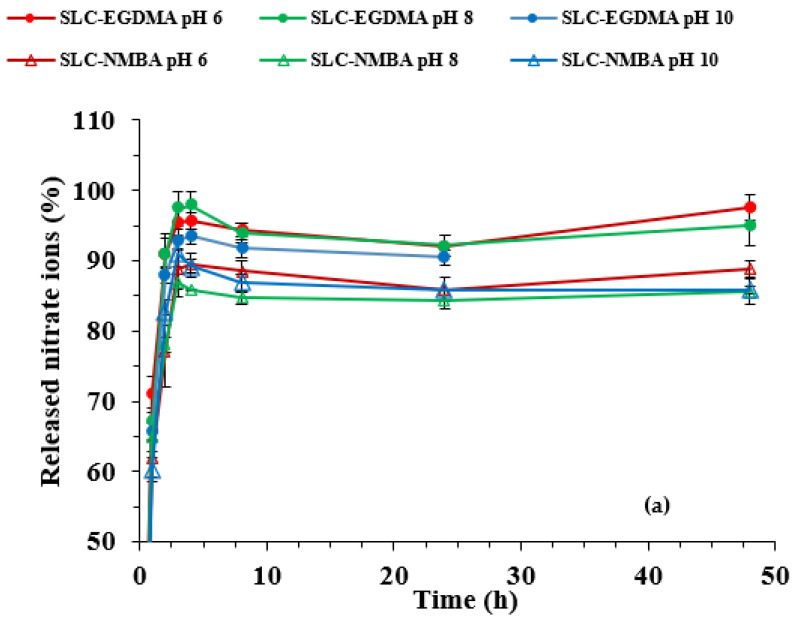

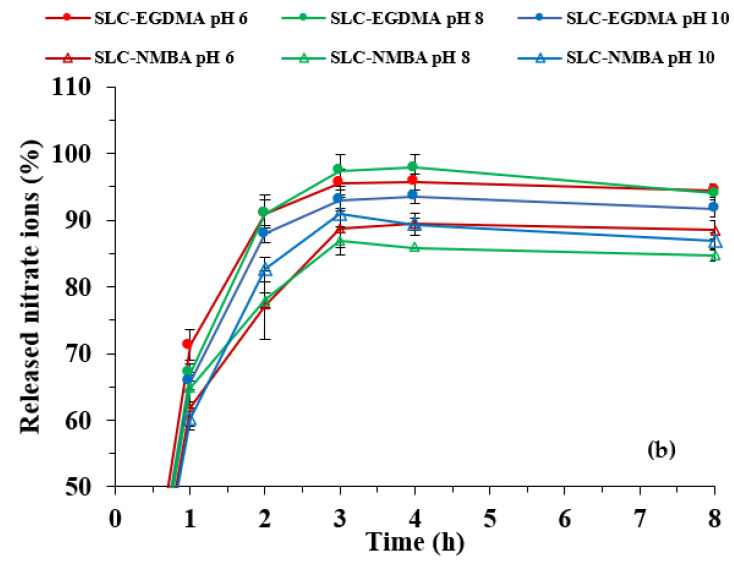
Release kinetic curves for SLC systems at pH 6, 8, and 10 (**a**) up to 48 h and (**b**) first hours.

Figure 6a shows the nitrate ion release kinetics curves up to 48 h at 25 °C in the selected pH range, whereas Figure 6b shows a magnification of the kinetics curves in the first 8 h for a better understanding.

Table 4 summarizes the nitrate release percentages (RN) corresponding to the time intervals where the highest release rate and maximum release occur in the pH range studied.

The kinetic curves show very high release rates in the first hour due to the dissolution of surface nitrate ions [30].

It is interesting to note that, as can be seen in the release kinetic curves obtained at pH 8 and 10, the nitrate release percentage declines slightly at the fourth hour for SLC-EGDMA and more clearly at the third hour for SLC-NMBA systems. According to Urbano et al. (2019) [30], this decrease in RN values is due to the reabsorption process of nitrate ions as they are dragged by water in the hydrogel swelling process.

According to the results showed in Figure 6b and Table 4, it could be seen that the SLC-EGDMA formulations showed higher increases in the release percentages from the first to the second hour (approx. 20%) and released the highest amount of nitrate ions. Finally, the nitrate ions release process was not affected by pH medium, except for SLC-EGDMA, which dissolved after 24 h at pH 10.

### 3.4. Diffusion Mechanisms

As can be seen in Table 5, high determination coefficients (R^2^ > 99%) were obtained from the linear fit of experimental data corresponding to the first 4 h of swelling to the Fick model, which indicates a high reliability of the data and that they follow first order kinetics. The water molecules penetration mechanism for both NMBA and EGDMA-containing polymeric systems, despite the pH of the medium, was found to be Non-Fickian or Anomalous since the values of n were between 0.66 and 0.97. This fact arises from the contribution of two concomitant processes: diffusion and relaxation of the polymer chains. These processes take place in the same time order, so that the predominance of one over the other will depend on the deviation from the Fickian behavior [53].

In relation to the parameter k, it was found that the values obtained for the systems containing NMBA were higher than their corresponding homologues with EGDMA. According to the criterion of Crank (1975) [54], higher values of k constant would indicate a higher degree of cross-linking of the NMBA system [53,54]. This is consistent with the lower values of the diffusion coefficient (D) obtained in these systems. On the other hand, the materials containing EGDMA showed lower k values (more open networks) and higher diffusion coefficients. All this coherence in the results obtained allows us to point out the good adaptation of the equation proposed by the present authors to the experimental results (R^2^ values higher than 99.48%). Finally, in relation to the influence of pH, no significant variations were observed in the parameters mentioned above.

As can be seen, the values of the coefficient of determination obtained (R^2^ > 93.15%) indicate a high reliability of the data and that the global swelling mechanism obeys a second order kinetics. This behavior is characteristic of processes where diffusion is controlled by the relaxation of the chains considering the global process [55,56].

The analysis of S_we_ (equilibrium water percentage) values confirmed all the above indicated. However, it is interesting to note the significant differences between the S_we_ values calculated from Schott’s model and those measured in the kinetic curves at 48 h for the EGDMA systems (Table 2 and Table 5). These large differences are justified by the fact that the kinetic curves of these systems did not reach equilibrium at 48 h. The analysis of the values of the apparent velocity constants, K_ap_, again revealed the faster swelling process in the systems containing EGDMA [57].

#### Release Kinetic

As can be seen in Table 6, the high values obtained for the coefficients of determination (R^2^) show a good reliability of the transport models in the release processes.

Similar results were obtained for the diffusion exponent (n) values and the kinetic constant (k), for all the SLCs, independently of the pH variation and type of crosslinking agent. On the one hand, n values (0.22 < n < 0.30) indicated an almost Fickian mechanism and that the diffusion of nitrate ions through the lattice structures of the SLCs is the limiting factor that regulates the release processes, that is, this phenomenon predominates over the relaxation process of the polymer chains because it occurs more slowly. On the other hand, the high k values indicated that the release of nitrate ions occurs in an uncontrolled manner, a phenomenon known as the burst effect [12,58,59]. This effect is mainly due to the immediate release of nitrate ions from the surface of the SLCs.

The high D values obtained from the model proposed by the authors for both polymeric systems confirmed the burst effect above indicated. On the other hand, the type of crosslinking agent as well as the pH of the medium did not clearly influence the nitrate ion release process.

In relation to Schott’s model, the results obtained for the equilibrium release percentage (RN) are very similar to the experimental data measured 48 h (Table 4). The values obtained for the apparent speed constant in the equilibrium (K_ap_) increases as the pH of the medium does for both polymeric systems. This increase could be justified by the greater openness of the networks produced by the increase in electrostatic repulsions as indicated above, being more evident at pH 10.

### 3.5. Behavior of Polymeric Systems in Dynamic Regime

The experiments in dynamic regime were carried out only with NMBA systems for several reasons: (i) they present a higher degree of cross-linking, (ii) high swelling capacity, and (iii) high stability in their structure independently of the pH of the medium. Thus, the potential utilization of NMBA systems as a reservoir or controlled release system in agricultural soils would not be conditioned by the pH of the medium.

Due to the great variation that can occur in the physicochemical properties of coconut fiber depending on how it is handled at source for the extraction of the usable product, in the Table 7 is shown those properties of greatest interest [60].

T-0 Test

No nitrate ions were detected in the leachates collected from the columns after passing 4 Vp (Vp = 270 mL) of distilled water.

On the other hand, experiments related to the study of the nitrate ion retention capacity of coconut fiber after passing a pulse of a solution containing 100 mg NO_3_^−^/L and its subsequent washing with 3 Vp of distilled water, showed that this material retained up to 25%.

T-1 Test

Table 8 shows the swelling percentages (H) together with their respective S_we_ values calculated by using Schott’s model for the H-NMBA and SLC-NMBA samples, for comparative purposes. Two aspects can be observed: on the one hand, H values are notably higher than those corresponding to S_we_ and, on the other hand, the swelling capacity of the H-system in the dynamic regime is much higher than that of the SLC-system in comparison with the slight difference observed in the static regime.

To understand these differences, it must be taken into account that, in dynamic studies, the samples are confined in beds containing a solid substrate (coconut fiber). Under these conditions, xerogels swelling will depend on the properties of the polymeric material, the properties of the coconut fiber–water–polymer mixture, and the confining stress [61]. Coconut fiber is a material characterized by the presence of macropores where water is retained by capillary forces that allow it to establish a wide humidity range in which water is readily available to plants [62]. For all these reasons, the high values of H in relation to those of S_we_ could be justified taking into account that the presence of coconut fiber induces a higher chemical affinity between capillary water molecules and polymeric chains. This fact, together with the osmotic swelling forces, generates a decrease in the free energy of the mixture, resulting in a further increase in its swelling capacity. This decrease in energy is opposed to the increase in free energy due to the elastic stretching of the polymeric network. The swelling reaches equilibrium when the stretching resistance forces equalize with the first [61,63,64].

According to the above indicated, the authors propose that the higher swelling observed in the polymeric systems confined in the coconut fiber columns is due to the higher chemical affinity of the capillary water molecules for the polymeric chains, which would favor their entry into the network by means of a diffusion mechanism. However, in the static regime, the global swelling mechanism according to Schott’s model would be controlled by the relaxation of the chains considering the global process.

The decrease observed in the swelling degree of the SLCs in comparison with their respective hydrogels would be justified, as indicated in the static experiments, based on the competition between the nitrate and potassium ions with the water molecules for the most hydrophilic functional groups of the structure and the shielding that these ions exert on the electrostatic repulsions present in them [65,66].

Finally, under the experimental conditions used—type of material (coconut fiber), depth at which the xerogel is placed (3 cm)—it is easy to understand that the confining forces acting on the hydrogel are practically negligible, which would justify that the samples maintain their initial shape and do not negatively affect their swelling capacity.

T-2 Test

The results obtained from the nitrate ion leaching study in columns using SLC-NMBA samples and commercial KNO_3_ are shown in Table 9. The data show that the use of KNO_3_ produces a high percentage of leaching in the first wash (92.42%) whereas nothing is detected in the third wash. However, when the SLC-NMBA formulation is used, the loss is slower and prolonged in time.

## 4. Conclusions

Conical geometry hydrogels based on acrylamide and itaconic acid (H-systems) have been synthesized together with controlled release systems containing potassium nitrate as active ingredient (SLC-systems) by a copolymerisation method and using two different crosslinking agents (EGDMA and NMBA). Nitrate ion incorporation was verified through FTIR spectroscopy, whereas SEM images and the use of the CPD technique showed the presence of inter-connected pores in their structures, which confers them as a big capillarity, converting them into superabsorbent materials.

Swelling studies showed an increment in swelling capacity (Sw) as pH increased for both H- and SLC-systems, the EGDMA systems showing the highest values due to their more flexible and open structures against the more rigid and closed polymeric network of NMBA, whose chemical structure allows the formation of additional cross-links.

The nitrate ions release process was not affected by pH medium, with high release rates in the first hour due to the dissolution of surface nitrate ions and with release percentages between 60–71%.

Swelling kinetics in these systems follows a first order kinetic, independently of the medium pH, and the penetration mechanism of the water molecules was non-Fickian or anomalous. The values of the diffusion coefficients (D) obtained using the equation proposed by the authors confirmed the information reported by the k parameter of the Fick model for both systems. Schott’s model indicates that the global swelling mechanism responds to second-order kinetics.

Regarding to the nitrate release kinetic, followed an almost Fickian mechanism, the high values of k (Fick model) and D (author’s model) parameters confirming an immediate release of nitrate ions (burst effect) from the surface of the SLCs.

Finally, the column studies performed with the NMBA systems showed on the one hand, higher swelling percentages for both H-NMBA and SLC-NMBA systems compared to those obtained in the batch experiments and, on the other hand, that the release of nitrate ions was slower and more gradual compared to commercial KNO_3_.

## 5. Future Perspectives

The results obtained in this study provide relevant information for a potential application of different polymeric matrices of itaconic acid and acrylamide that are capable of effectively regulating the supply of potassium nitrate to different types of soils, in such a way that the needs of a specific crop could be met throughout its growth cycle. Besides this, the use of these polymers will increase the water retention capacity of the soil, thus favouring water saving and better plant development. Therefore, these systems constitute a promising alternative to the use of conventional fertilizers and contribute to the creation of a more sustainable agriculture. However, data from batch experiments suggest the need for slowing down the process of nitrate ion release at the first stages. From the author’s perspective, attention should be given to further research to optimise the composition of hydrogels to deliver the adequate amount of fertilizer, combining maximum effectiveness with minimum doses.

## Figures and Tables

**Table 1:** Diffusion Mechanism classification.

n	Diffusion Mechanism
n < 0.5	Almost Fickian
n = 0.5	Fickian
0.5 < n < 1	Non-Fickian or Anomalous
n = 1	Case II
n > 1	Supercase II

**Table 2:** Swelling percentages at 48 h and linear initial swelling velocity at different pHs ^a^.

Polymeric System	pH	S_w_ (%)	V_i_ (h^−1^)	R^2^
H-NMBA	2	423	0.45	99.15
	4	927	0.9	99.46
	6	1062	1.18	98.95
	8	1148	1.22	98.95
	10	1126	1.21	98.74
H-EGDMA	2	1052	0.71	99.97
	4	2672	1.67	99.96
	6	3550	2.36	99.68
	8	4142	2.46	99.82
	10	---	2.76	99.95

^a^ the error was < 0.005.

**Table 3:** Swelling percentages at 48 h and linear initial swelling velocity at different pHs ^a^.

Polymeric System	pH	S_w_ (%)	V_i_ (h^−1^)	R^2^
SLC-NMBA	6	1017	0.94	99.73
	8	1078	0.99	99.69
	10	1185	1.04	99.72
SLC-EGDMA	6	1809	1.54	99.55
	8	2236	1.59	99.62
	10	---	1.79	99.83

^a^ the error was <0.005.

**Table 4:** Nitrate Release percentages at different time intervals and pH ^a^.

Polymeric System	pH	RN 1 h (%)	RN 2 h (%)	RN 48 h (%)
SLC-NMBA	6	61.9	77.2	88.8
	8	64.9	78.0	85.6
	10	60.2	82.6	85.8
SLC-EGDMA	6	71.3	91.0	97.6
	8	67.1	91.0	94.9
	10	65.8	88.0	-----

**^a^** The error was <0.005.

**Table 5:** Parameters related to diffusion mechanism for the Fick, Authors, and Schott Models ^a^.

		Fick Model			Authors Model		Schott Model		
Polymeric System	pH	n	k	R^2^ (%)	D (cm^2^/s)	R^2^ (%)	S_we_ (%)	K_ap_ (h^−1^)	R^2^ (%)
H-NMBA	6	0.79	0.34	99.74	0.09	99.48	1190	2.36	99.80
	8	0.76	0.36	99.80	0.08	99.61	1285	2.58	99.82
	10	0.71	0.38	99.77	0.07	99.56	1242	2.78	99.93
SLC-NMBA	6	0.70	0.38	99.68	0.09	99.72	1160	1.91	99.83
	8	0.66	0.40	99.56	0.08	99.63	1220	2.14	99.84
	10	0.70	0.38	99.76	0.09	99.78	1355	1.98	99.96
H-EGDMA	6	0.97	0.27	99.82	0.13	99.74	4808	2.87	99.82
	8	0.90	0.29	99.96	0.12	99.95	5682	3.12	99.93
	10	0.91	0.28	100	0.12	100	-----	-----	-----
SLC-EGDMA	6	0.77	0.34	99.92	0.14	99.93	2123	2.70	99.94
	8	0.74	0.36	99.94	0.13	99.94	2681	2.69	99.88
	10	0.75	0.35	99.94	0.13	99.89	-----	-----	-----

^a^ The error was <0.005.

**Table 6:** Parameters related to nitrate diffusion mechanism for the Fick, Authors, and Schott Models.

		Fick Model			Authors Model		Schott Model		
Polymeric System	pH	n	k	R^2^ (%)	D (cm^2^/s)	R^2^ (%)	R_N_ (%)	K_ap_ (h^−1^)	R^2^ (%)
SLC-NMBA	6	0.28	0.70	96.46	4.69 × 10^−3^	95.30	88.74	4.09	99.97
	8	0.22	0.77	93.68	1.12 × 10^−3^	92.15	85.72	6.10	99.99
	10	0.30	0.70	89.26	6.26 × 10^−3^	86.66	85.90	35.71	99.99
SLC-EGDMA	6	0.22	0.77	88.64	1.51 × 10^−3^	86.96	97.16	3.44	99.92
	8	0.28	0.71	89.77	5.99 × 10^−3^	87.84	94.76	7.18	99.97
	10	0.26	0.73	88.82	4.08 × 10^−3^	86.91	90.97^*^	16.21 *	99.96 *

* Data collected up to 24 h.

**Table 7:** Coconut fiber physico-chemical properties.

Physico-Chemical Property	
Total pore space (%)	96.30
Aeration capacity (% vol)	32
Apparent Density (g/cm^3^)	0.06
Real Density (g/cm^3^)	1.52
Water retention capacity (mL/L)	523
pH	5.98
Electric Conductivity (dS/m)	3.52
Organic mass (%)	92.07

**Table 8:** Swelling Percentages obtained in T-1 test.

Polymeric System	H (%)	S_we_ (%)
H-NMBA	2646	1190
SLC-NMBA	1931	1160

**Table 9:** Nitrate leached percentage obtained in the dynamic study ^a^.

Leachate	KNO_3_	SLC-NMBA
	R_N_ (%)	R_N_ (%)
1	92.42	28.67
2	12.38	55.86
3	0	3.37
4	0	0
Total	104.8	87.91

^a^ The error was <0.005.

## Data Availability

The data presented in this study are openly available in http://repositorio.ual.es (accessed on 28 February 2023) at http://hdl.handle.net/10835/10862 (accessed on 28 February 2023).
